# Vitamin D and inflammation

**DOI:** 10.4161/19381980.2014.983401

**Published:** 2015-01-29

**Authors:** John J Cannell, William B Grant, Michael F Holick

**Affiliations:** 1 Vitamin D Council; San Luis Obispo, CA USA; 2 Sunlight, Nutrition, and Health Research Center; San Francisco, CA USA; 3 Department of Medicine; Section on Endocrinology, Nutrition and Diabetes, Vitamin D, Skin and Bone Research Laboratory; Boston University Medical Center; Boston, MA USA

**Keywords:** cardiovascular disease, C-reactive protein, cytokines, inflammation, randomized controlled trials, tumor necrosis factor-alpha, vitamin D

## Abstract

Several studies found an inverse relationship between 25-hydroxyvitamin D [25(OH)D] and markers of inflammation. A controversy exists as to whether vitamin D lowers inflammation or whether inflammation lowers 25(OH)D concentrations. Certainly 25(OH)D concentrations fall after major surgery. However, is this due to inflammation lowering 25(OH)D or is 25(OH)D being metabolically cleared by the body to quell inflammation. We searched the literature and found 39 randomized controlled trials (RCT) of vitamin D and markers of inflammation. Seventeen found significantly reduced inflammatory markers, 19 did not, one was mixed and one showed adverse results. With few exceptions, studies in normal subjects, obesity, type 2 diabetics, and stable cardiovascular disease did not find significant beneficial effects. However, we found that 6 out of 7 RCTS of vitamin D_3_ in highly inflammatory conditions (acute infantile congestive heart failure, multiple sclerosis, inflammatory bowel disease, cystic fibrosis, SLE, active TB and evolving myocardial infarction) found significant reductions. We found baseline and final 25(OH)D predicted RCTs with significant reduction in inflammatory markers. Vitamin D tends to modestly lower markers of inflammation in highly inflammatory conditions, when baseline 25(OH)D levels were low and when achieved 25(OH)D levels were higher. Future inquiries should: recruit subjects with low baseline 25(OH)D levels, subjects with elevated markers of inflammation, subjects with inflammatory conditions, achieve adequate final 25(OH)D levels, and use physiological doses of vitamin D. We attempted to identify all extant randomized controlled trials (RCTs) of vitamin D that used inflammatory markers as primary or secondary endpoints.

## Abbreviations

CRP: C-reactive protein
CXCL9: CXC chemokine ligand 9
E-selectin: ESR, erythrocyte sedimentation rate, F1+2, prothrombin fragment 1+2
IFG: impaired fasting glucose
IL: interleukin
MCP-1: monocyte chemoattractant protein-1
NGAL: neutrophil gelatinase-associated lipocalin
NGT: normal glucose tolerance
ns: not stated
PAI-1: plasminogen activator inhibitor-1
sICAM-1: soluble intracellular adhesion molecule-1
sTNF-R2: soluble tumor necrosis factor α receptor type 2
TAT: thrombin antithrombin complex
TGF-β: transforming growth factor-β
TNF-α: tumor necrosis factor-α

## Introduction

Everything from depression^1^ to cardiovascular disease^2^ to cancer^3^ and autoimmune disorders^4^ is theorized as having inflammation as a core etiological factor. In measuring that inflammation, there are general markers of inflammation, such as C-reactive protein (CRP) and erythrocyte sedimentation rate (ESR), but most research on inflammatory markers are focused on the cytokines.^5^ In fact, modulating inflammatory cytokines is now a mainstay of treatment in a number of diseases.^6^

The relationship between vitamin D and inflammation has been controversial. Some hypothesize that inflammation reduces 25(OH)D concentration,^7-9^ while others hypothesize increasing vitamin D status reduces inflammation.^10-12^ Those who hypothesize the first explanation, that inflammation lowers 25(OH)D concentration, theorize that inflammation lowers serum 25(OH)D via oxidative stress resulting in the oxidative catabolism of 25(OH)D. They hypothesize that an oxidative environment reduces 25(OH)D by interfering with key vitamin D metabolizing enzymes, disturbing the liver's biosynthesis of 25(OH)D, thus lowering 25(OH)D concentration. However, this is a difficult theory to disprove.

While it has been shown that 25(OH)D concentrations decline after major surgery, a fact used to support that inflammation lowers 25(OH)D concentration hypothesis, to our knowledge, the question of metabolic clearance has not been discussed. However, it is clear that 25(OH)D concentration decline after major surgery.^13-15^ Is that because inflammation lowers 25(OH)D concentration or because 25(OH)D is metabolically cleared by the body as it utilizes 25(OH)D by converting it to 1,25(OH)_2_D_3_ to modulate inflammation? That is, perhaps the body uses 25(OH)D in an effort to heal itself, thus lowering 25(OH)D concentration.

As far as the second contention, that vitamin D decreases inflammation, certainly in vitro studies indicate 1,25(OH)_2_D_3_ has potent anti-inflammatory properties.^16-18^ Animal studies also indicate 1,25(OH)_2_D_3_ and its analogs are effective anti-inflammatories.^19-^^21^ The mechanisms by which 1,25(OH)_2_D_3_ reduces inflammation are multiple. 1,25(OH)_2_D_3_ affects both the innate and adaptive immune systems; the overall effect is a switch from the more inflammatory T-helper 1 (Th1)/Th17 response to the less inflammatory Th2/Treg profile.^22^ In vitro, these effects result in decreased production of pro-inflammatory markers such as: tumor necrosis factor α (TNF-a), interferon-gamma (IFN-g), interleukin (IL)-2, IL-12, IL-17 and IL-21 but with increased production of anti-inflammatory cytokines such as IL-10.^23^

Some cross-sectional studies have shown a relationship between 25(OH)D and markers of inflammation. In the largest report, Amer and Qayyam studied more than 15,000 subjects from the National Health and Nutrition Examination Survey (NHANES) 2001–2006.^24^ The subjects were divided into low and high 25(OH)D concentration with a cutoff of 53 nmol/L. In subjects with a 25(OH)D < 53 nmol/L, serum 25(OH)D was inversely associated with CRP (β = − 0.34; *P* < 0.001) but there was no significant association between serum 25(OH)D and CRP in those with serum 25(OH)D greater than 53 nmol/L (β = − 0.05; *P* = 0.07).

CRP is not the only inflammatory marker that has been identified as having cross sectional relationships to circulating 25(OH)D. Bellia et al. studied the relationship between serum 25(OH)D and several markers of inflammation in 147 morbidly obese subjects.^25^ Mean 25OHD was 65 nmol/L. In this group, serum 25(OH)D was significantly inversely correlated not only with CRP (r = − 0.31; P = 0.043), but also with IL-6 (r = − 0.50; P = 0.003), and TNF-α (r = − 0.61; *P* = 0.001).

It's important to note that 25(OH)D is a marker for sunlight exposure. Also, ultraviolet radiation has effects on the immune system that are independent of vitamin D.^26,27^ If sunlight, in a path or pathways entirely independent of vitamin D, explains the associations of inflammatory markers and 25(OH)D concentration, then RCTs of vitamin D in markers of inflammation will not find beneficial effects. It is also possible that sunlight and vitamin D may have complimentary effects on inflammation.

The observation that higher 25(OH)D is associated with decreased concentration of inflammatory markers suggests that local autocrine production of 1,25(OH)_2_D_3_ is binding to genes down-regulating pro-inflammatory cytokines and up-regulating anti-inflammatory ones.

If inflammatory conditions decrease 25(OH)D concentration through metabolic clearance, and the body utilizes vitamin D to help heal and modulate inflammation, it may mean that higher 25(OH)D concentration may be beneficial in inflammatory conditions and thus in treating some inflammatory diseases. In support of such a contention, is the finding that higher 25(OH)D concentration are associated with faster recovery from induced muscle injury.^28^ Furthermore, a RCT of 28 healthy adults found 100 μ day of vitamin D_3_ enhanced the recovery in peak isometric force after a muscle-damaging event (*P* < 0.05).^29^ In that study, supplemental vitamin D_3_ attenuated (*P* < 0·05) the immediate and delayed (2 day, 3 day and 7 day) increase in circulating biomarkers of muscle damage.

In its endocrine role 1,25(OH)_2_D_3_ helps maintain the calcium economy. 1,25(OH)_2_D_3_ is derived from a cholesterol precursor metabolite to 1,25(OH)_2_D_3_ (7-dehydrocholesterol), which, when exposed to sunlight, is converted to vitamin D_3_. Once formed, vitamin D_3_ enters the circulation and is sequentially hydroxylated first in the liver to 25(OH)D and then in the kidney and various tissues to 1,25(OH)_2_D_3_. In its endocrine role, the seco-steroid 1,25(OH)_2_D_3_ is secreted into the blood by the kidneys and affects downstream target tissues by interacting with the nuclear vitamin D receptors (VDR) to help maintain the calcium economy. Unlike other anti-inflammatories, e.g. corticosteroids, exogenous 1,25(OH)_2_D_3_ cannot be used as an anti-inflammatory in pharmacological doses because of dose dependent hypercalcemia.^30^

However, a growing literature suggests 1,25(OH)_2_D_3_ also has autocrine (inside the cell) steroid actions.^31^^,^ ^32^ What is theorized for 1,25(OH)_2_D_3_s autocrine actions is that 25(OH)D is delivered to cells via the blood, transported across the cell membrane by both passive and active transport,^33^ and metabolized into 1,25(OH)_2_D_3_ by intracellular mitochondrial 25(OH)D 1-hydroxylase (CYP27B1). Under physiological conditions, serum 25(OH)D concentrations are a thousand fold higher than serum 1,25(OH)_2_D_3_ concentrations. What is not known is the relative membrane transportation rate, both via active and passive mechanisms, of 25(OH)D and 1,25(OH)_2_D_3_. If their membrane transportation rates are the same, this may mean that intracellular 25(OH)D concentration are much higher than intracellular 1,25(OH)_2_D_3_ concentrations.

Because the renal production of 1,25(OH)_2_D_3_ is tightly regulated, increasing vitamin D_3_ intake does not result in an increase in serum 1,25(OH)_2_D_3_ concentration.^34^ However, the administration of increasing doses of vitamin D_3_ will increase the amount of serum 25(OH)D available for trans-membrane transport without changing the amount of serum 1,25(OH)_2_D_3_ available for trans-membrane transport.

Thus it is theorized, but not currently discoverable for technical reasons, that the intracellular concentration of autocrine-produced 1,25(OH)_2_D_3_ may greatly exceed the intracellular concentration that can be reached by administering exogenous 1,25(OH)_2_D_3_ as a pharmaceutical. That is, physiological doses of vitamin D_3_, i.e., less than 10,000 IU/d, and the resultant increased serum concentration of 25(OH)D, may result in increased intracellular concentration of 1,25(OH)_2_D_3_, concentration not achievable either from the controlled renal production of 1,25(OH)_2_D_3_ or the exogenous administration of 1,25(OH)_2_D.

There are unresolved technical problems with attempting to prove this theory, as one cannot yet accurately measure intracellular concentration of 1,25(OH)_2_D_3_ achieved in an autocrine manner compared with the intracellular concentration achieved when 1,25(OH)_2_D_3_ is delivered in an endocrine manner. However, when vitamin D_3_ is given, the resultant autocrine intracellular 1,25(OH)_2_D_3_ concentration may be much higher than that achieved by directly administering exogenous 1,25(OH)_2_D_3_.

Therefore, the possibility exists that physiological doses of vitamin D and the resultant higher serum 25(OH)D concentration may ultimately achieve a high intracellular 1,25(OH)_2_D_3_ level. If so, vitamin D, like the glucocorticoids, may be clinically useful when used in physiological or perhaps pharmaceutical doses as there are between 1,000 and 13,000 VDR-specific genomic binding sites, some of them linked to inflammation.^35^ When healthy adults received 2000 IU vitamin D_3_ daily for 3 months 291 genes in their immune white blood cells were substantially influenced. These genes were related to as many as 80 metabolic processes including inflammatory pathways.^36^

Any review of the RCT of vitamin D and markers of inflammation is complicated because the studies are so heterogeneous. First, there are studies of various disease states, some much more inflammatory than others. For example, active TB or active lupus is surely more inflammatory than is, for example, obesity or type-2 diabetes. Likewise, subjects suffering an acute myocardial infarction are certainly undergoing a much more inflammatory process than are those who have stable cardiovascular disease. If vitamin D is anti-inflammatory, we assume it will be more likely to show an effect in highly inflammatory diseases.

Also, there are multiple markers of inflammation; it is unlikely that — if vitamin D affects these markers — it would do so to the same degree with each marker. That is, if vitamin D modulates inflammation, it seems likely that some inflammatory markers would be more responsive to vitamin D than others.

Other factors causing heterogeneity include baseline 25(OH)D, final 25(OH)D, baseline markers of inflammation, dose of vitamin D used, type of vitamin D used (D_2_ or D_3_) and duration of treatment.

In any definitive RCT of vitamin D used as a drug to modulate inflammation, final achieved 25(OH)D concentration must be adequate not to miss a treatment effect although no one knows what adequate is. Certainly, as we will see, RCTs of vitamin D and markers of inflammation using physiological doses of vitamin D are rare.

We somewhat arbitrarily categorized physiological doses of vitamin D as the doses theorized to optimize all vitamin D requirements, which are currently under debate. If nature is any guide as to natural 25(OH)D concentration, 25(OH)D concentration of lifeguards range from 100 to 200 nmol/L.^37^ Modern day equatorial hunter-gatherers have mean 25(OH)D concentration of 115 nmol/L.^38^ Such concentrations require total vitamin D inputs of more than 125 μg/day. For example, when administered 25, 100, or 250 μg/day for 20 weeks during the winter, healthy men utilized approximately 125 μg of total vitamin D input/day just to maintain baseline concentration of around 75 nmol/L.^39^ Therefore, one can argue that physiological doses in sun-deprived individuals needed to maintain natural 25(OH)D concentration of 50 nmol/L are at least 125 μg/day.

In order to accurately study the full effect of vitamin D in inflammation, it is likely that baseline 25(OH)D concentration must be low to begin with in order to see an effect, at least when using physiological doses of vitamin D. For example, as CRP is only inversely associated with 25(OH)D when 25(OH)D concentrations are below 53 nmol/L, it is unlikely that a randomized controlled trial of vitamin D will lower CRP when baseline 25(OH)D concentration are >53 nmol/L.

Also, it is possible that vitamin D_3_ and vitamin D_2_ may have different effects on markers of inflammation. For example, a RCT of vitamin D_2_ and muscle damage in 28 subjects found vitamin D_2_ increased, not decreased, biomarkers of muscle injury compared to placebo.^40^

Finally, to show an effect, the markers of inflammation probably need to be elevated at baseline. That is, it is unlikely that vitamin D will lower markers of inflammation that are relatively low to begin with.^41^

## Findings

Thirty-nine RCTs fit our qualifications. **Table 1** lists each of the 39 studies, the population studied, the medical condition studied, age, number of subjects studied, biomarkers of inflammation used, baseline and final 25(OH)D concentration dose used, duration of treatment and outcome.

**Table 1.** Parameters of vitamin D RCTs

Reference	Population location	Condition	Age (yrs)	N	Biomarker	Baseline 25(OH)D (nmol/L)	Achieved 25(OH)D (nmol/L)	Vitamin D dose (μg/d)	Duration (wks)	Outcome (*P* value)
^42^Gepner, 2012	Wisconsin	Healthy	64 ± 3	119	CRP	75.8	120	62.5	17	CRP, 0.97
^43^Pittas	Boston	Healthy	71	314	CRP, IL-6	71.2 vs. 81·2*	102.4 vs 73·4	500 mg/d Ca + 17.5/d	156	CRP, 0.87; IL-6, 0.78
^44^Von Hurst, 2010	New Zealand S. Asian women	Healthy	42 ± 10	81	CRP,	21.0	80	100	26	CRP, 0.05
^45^Bjorkman, 2009	Finland	Long-term care patients	85 ± 8	218	CRP	23.8	72.6	30	26	No change
^46^Barnes, 2011	Ireland	Community	71 ± 4	211	CRP, IL-6, IL-10, TNF-α	55	74	15	22	p > 0.05
^47^ Wood, 2012	UK	Healthy postmenopausal women	64 ± 2	305	CRP, ICAMs-1, IL-6	32.4	65.5, 75.3	25 vs 10	52	CRP, 0.73; .ICAMs-1, 0.67; IL-6, 0.84
^48^Chandler, 2014	Massachusetts	Community, African-Americans	51	328	CRP, IL-6, IL-10, sTNF-R2	38	115	25, 50, 100	13	CRP, 0.91; IL-6, 0.84; IL-10, 0.40; sTNF-R2, 0.35
^49^Bischoff-Ferrari, 2012	Zurich	Healthy women	63 ± 8	20	Eotaxin, IL-12, MCP-1, MIP-1β	35.5	77.5	20	17	Eotaxin, 0.0002; IL-12, <0.001; MCP-1, 0.01; MIP-1β, 0.01
^50^Asemi, 2013	Iran	Pregnant, healthy	25 ± 4	48	CRP, TAC,	44.5	53.8	10	9	CRP, 0.01; TAC, 0.002
^51^Jorde, 2010	Norway	Community, obese	21–70	324	CRP, ICAM-10, IFN-c, IL-2, IL-4, IL-5, IL-10, IL-12, IL-13, IL-17,	57	101, 134	500 or 1000/wk	52	p > 0.05
^52^Beilfuss, 2012	Norway	Overweight, obese	50	332	CRP, IL-6, TNF-α	54.3	99	50, 100/wk	52	Elevated CRP <0.05; IL-6, 0.08; others >0.05
^53^Belenchia, 2013	Missouri	Obese adolescents	14 ± 3	35	CRP, IL-6, TNF-α	49.0	98.0	100	26	CRP, IL-6, TNF-α, >0.05
^54^ Carrillo, 2013	Indiana	Overweight, obese	26 ± 5	23	CRP, IL-6, TNF-α	52	83.5	91	12	CRP, >0.5; IL-6, >0.5; TNF-α, >0.5
^55^Zittermann, 2009	Germany	Overweight, obese	48 ± 10	165	CRP, IL-6, TNF-α (treatment x time)	30.0	85.5	83	52	CRP, 0.48; IL-6, 0.12; TNF-α, 0.049
^56^Mason, 2104	Washington	Obese undergoing weight loss	60 ± 5	120	CRP	53.5	87.5	50	52	CRP, 0.03
^57^Wamberg, 2013	Denmark	Obese	40 ± 8	52	CRP, IL-6	34.5	110.2	175	26	p > 0.05
^58^Hopkins, 2011	Georgia	Colorectal adenoma patients	60 ± 8	92	Combined inflammation Score	52.5	72.5	20	26	P = 0.003
^59^Rahimi-Ardabili, 2012	Iran	PCOS patients	27 ± 5	50	CRP	17.3	58.5	1250/20 days	8	CRP, 0.68
**^60^**Sinha-Hikim, 2014	California,	Pre-diabetics, African-American and Latino,	52 ± 7	80	CRP, IL-6, TNF-α,	54.8	175	305	52	CRP, 0.43; IL-6, 0.67; TNF-α, 0.43
^61^Yiu, 2013	Hong Kong	T2DM patients	65 ± 9	100	CRP	52.8	139.6	125	12	CRP, 0.72
^62^Breslavsky, 2013	Israel	T2DM patients	66 ± 10	47	CRP	29.5	44	25	52	CRP, 0.60
^63^Grimnes, 2011	Norway	Community	52 ± 9	94	CRP	42.2	142.7	1000/wk	26	CRP, 0.64;
^64^Shab-Budarm 2012	Iran	T2DM patients	30–60	100	E-selectin	38.0	72	25	12	Decreased, 0.035
^65^Kampman, 2014	Denmark	T2DM patients	62 ± 4	16	CRP, IL-6, IL-10, TNF-α	31.0	104	140	13	CRP, >0.05; IL-6, 0.41; IL-10, 0.40; TNF-α, 0.63
^66^Bucharles, 2011	Brazil	Hemodialysis patients, renal failure	59 ± 15	30	CRP, IL-6	45.3	101	1250, then 500/wk	26	CRP, 0.04; IL-6, 0.02
^67^Alvarez, 2013	Georgia	Early chronic kidney disease	63 ± 10	46	IL-6, IP-10, MCP-1, TNF-α,	67.5	193	1250/wk	12	MCP-1, 0.02; others >0.05
^68^Sokol, 2012	New York	cardiac catheterization patients	55 ± 10	90	IL-6, IL-12, CRP	32.5	100	1250/wk D_2_	12	IL-6, 0.94; IL-12; 0.72; CRP, 0.19
^69^Stricker, 2012	Switzerland	PAD patients	73 ± 9	62	inflammation parameters	41	61	2500 once D_2_	4	TAT, 0.94; F1+2, 0.54; PAI-1, 0.47; CRP, 0.61
^70^Witham, 2010	Scotland	Systolic heart failure patients	79 ± 6	105	TNF-α,	20.5	40	2500 at baseline, 10 wks D_2_	20	TNF-α, 0.85
^71^Schleithoff, 2006	Germany	CHF patients	57 ± 4	123	CRP, IL-10, TNF-α	38.0	103	50	39	CRP, 0.25; IL-10, 0.04; TNF-α, 0.006
^72^Longenecker, 2012	Georgia	HIV patients	47 ± 8	45	CRP, IL-6	22.5	35	100	12	CRP, 0.29; IL-6, 0.01
^73^Mahon, 2003	New York	Multiple sclerosis patients	ns	39	TNF-α, IFN-g, IL-2, IL-13, TGF-β	42.5	70	25	26	Only TGF-β improved, (no *P* values)
^74^Pappa, 2014	Boston	Children with IBD	15 ± 3	63	Number of those with elevated CRP, IL-6, ESR	82.5 vs 70·0	75-87 vs 69-74	25 in fall, 50 in winter vs. 10* (D_2_)	52	CRP, 0.05; IL-6, 0.04; ESR 0.10
^75^Grossmann, 2012	Georgia	Cystic fibrosis patients	25 ± 16	30	IL-1β, IL-6, IL-8, IL-10, IL-18-iBP, TNF-α	76.5	92	6250 once	12	IL-6, 0.11; IL-8, 0.01; TNF-α, 0·005; others >0.10
^76^Shedeed, 2012	Egypt	Infants, CHF	10 ± 5 mos	80	IL-6, IL-10, TNF-α	33.5	82.2	25	12	All improved, P < 0.001
^77^Abou-Raya, 2013	Egypt	Systemic lupus erythematosus patients	39 ± 6	267	IL-1, IL-6, IL-18, TNF-α,	19.9	49.8	94.5	52	All improved, p < 0.05
^78^Hansen, 2014	Wisconsin	Patients with RA	45–55	22	TNF-α	62.5	75	1250/2 wks (D_2_)	52	TNF-α Increased, p = 0.04
^79^Arnson, 2013	Israel	ACS patients	60 ± 13	50	CRP, IL-6, IL-8, TNF	46.7 vs 45.6	62.3 vs 57.2	100	5 days	CRP, 0.03; IL-6, 0.05; IL-8, 0.10; TNF, 0.16
^80^Coussens	UK	TB patients	32 (24–42)	146	ESR, CRP, 9 cytokines, 7 chemokines	23.8	108	2500 on day 7, 14, 28 and 42	8	CRP, 0.007; ESR, <0.001; CXCL9, <0.001; IL-10, 0.002

The 39 RCT were heterogeneous with respect to inflammatory processes studied, markers of inflammation studied, baseline concentration of markers of inflammation, baseline concentration of 25(OH)D, final concentration of 25(OH)D, and dose, duration and type of vitamin D used.

Of those 39 RCTs, 19 showed no effect, 19 showed a significant beneficial effect (on at least one marker), one study showed mixed results (IL-6 improved, TNF was unchanged, and CRP worsened), and a RCT in rheumatoid arthritis showed vitamin D_2_ worsened TNF-α. Of the 19 studies without beneficial effects, 18 were in mildly inflammatory conditions. Of the 19 studies with significant beneficial effects, all were either in highly inflammatory conditions, used doses of vitamin D >20 μg/day and had baseline CRP >3.

The results of the trials in **Table 1** can be ordered by baseline and achieved 25(OH)D concentration and the number of significant and insignificant findings tabulated (**Table 2**). Trials involving vitamin D_2_ were not included. Given the relatively small number of trials that dealt with inflammation using vitamin D_3_, 34, it was deemed appropriate to divide the data into 2 groups for each ranking. Appropriate breaks appeared to be 51.9 nmol/L for baseline 25(OH)D and 83 nmol/L for achieved 25(OH)D. The group with the lower 25(OH)D concentrations was considered the treatment group, while the other group was considered the control group. The findings from each study were apportioned between significant and not significant at the p=0.05 level, then totaled. A calculator for relative risk with 95% confidence intervals was used.^81^

**Table 2.** Relative risk of a vitamin D_3_ trial finding a significant reduction in inflammation biomarkers based on baseline and achieved 25(OH)D concentrations

Order	Range (nmol/L)	N	N Successful	N Unsuccessful	Relative Risk of Successful Trial
Baseline 25(OH)D concentration	17.3–46.7	22	10.2	10.8	
	49.0–76.5	12	3.5	9.5	
Totals		34	13.7	20.3	1.40 (95% CI, 0·84–2·34)
					
Achieved 25(OH)D concentration	35–82.2	15	8.2	6.8	
	83.5–187	19	5.5	13.5	
Totals		34	13.7	20.3	1.79 (95% CI, 0.84–3.79)

CI, confidence interval; N, number of trials.

For this set of vitamin D RCTs related to inflammation biomarkers, the relative risk for a significant outcome based on baseline 25(OH)D concentrations divided between 46.7 and 49.0 nmol/L was 1.40 (95% CI, 0·84–2·34 (see **Table 2**). The relative risk for a significant outcome based on achieved 25(OH)D concentrations divided at 83 nmol/L was 1·79 (95% CI, 0·84–3·79), with lower achieved 25(OH)D concentrations being more likely to result in a significant outcome. The likely reason that lower rather than higher achieved 25(OH)D concentrations were more likely to be associated with a significant outcome is that they were more likely to be associated with lower baseline 25(OH)D concentrations. 25(OH)D-health outcome relations change rapidly for 25(OH)D concentrations below 50 nmol/L, then change slowly for higher values.^82,83^

These results are consistent with the guidelines for clinical trials for nutrient effects proposed by Robert Heaney.^84^ Two important guidelines were that those enrolled in the trials should have values of the nutrient marker of interest at the lower end of the nutrient-health outcome relationship, and then be given in sufficient enough amounts to raise the marker to the upper end of the relationship.

The implication of this finding is that unless those enrolled in vitamin D trials have low 25(OH)D concentrations, the trails are unlikely to find significant beneficial effects. This finding helps explain why few vitamin D RCTs have found significant beneficial effects, as noted by Autier ^7^ and others. This finding also suggests that the major vitamin D trials underway, such as VITAL,^85^ will be unlikely to find significant beneficial effects except in those participants with low baseline 25(OH)D concentrations.

## Discussion

As our review suggests, the effectiveness of vitamin D in lowering markers of inflammation appears to depend mainly on the disease state studied and baseline 25(OH)D concentrations. Some conditions are more inflammatory than others. For example, active TB or evolving myocardial infarction is surely more inflammatory than is, for example, obesity. Likewise, subjects with SLE are certainly undergoing more inflammation than are subjects with stabletype-2 diabetes.

We found that 7 out of 8 RCTS of vitamin D_3_ in highly inflammatory conditions (acute infantile congestive heart failure, multiple sclerosis, inflammatory bowel disease, cystic fibrosis, SLE, active TB and evolving myocardial infarction) found significant beneficial effects. One study in rheumatoid arthritis showed an adverse effect but that study used vitamin 1250 μg/(2 weeks) of D_2_ not D_3_.

The most meticulous of the studies in highly inflammatory conditions (active TB), Coussens et al,^81^ started with baseline CRP of 62.5 mg/L in the treatment group and it fell to 15.5 mg/L while the initial CRP in the placebo group was 62 mg/L initially and it fell to 19 mg/L after 8 weeks of anti-tubercular treatment (*P* ≤ 0.0072). Although highly significant, the effect on CRP was modest at best.

To put these findings in context, it is useful to compare vitamin D's anti-inflammatory actions to those of a corticosteroid.^86^ When 12.5 mg/day of prednisone/day was used to treat polymyalgia rheumatic, ESR declined from 60 to 20 mm/hour and CRP declined from 30 to 5 mg/dl within one week. However, prednisone in such doses is not free from serious side effects while physiological doses of vitamin D appear to be.

In looking at the conditions studied, in normal subjects, 7of the 8 studies found no beneficial effects including 2 studies using low physiological doses of vitamin D_3_ (63 μg/day and 100 μg/day). The only RCT with beneficial effects in normal subjects was a small study that used calcidiol [25(OH)D] instead of cholecalciferol (vitamin D_3_) as the intervention. The dose of 25(OH)D used were supra-physiological, equivalent of up to 200 μg/day of vitamin D_3_.^87^

Four of the 7 studies in obesity did not find beneficial effects, one was mixed, and the 2 RCTs with beneficial effects were of marginal significance or only significant among high compliers. It does not appear vitamin D significantly lowers markers of inflammation in obesity.

As we said, such studies are complicated by the wide variation in doses of vitamin D used as well as the method of administration. Six studies used monthly bolus dosing or a greater time interval and 2 of them found significant beneficial effects. As far as dose, some studies attempted to modulate inflammatory markers using doses as low as 200 IU/day of vitamin D_3_, while others used 15,000 μg every month, and one used 20 μg/day of calcidiol, which, as noted above, may be equivalent of 200 μg/day of vitamin D_3_.

In the 6 studies that used vitamin D_2_ as treatment, 4 of 6 showed no effect, one found a beneficial effect, and one showed a detrimental effect. That is, only one of the 6 studies of vitamin D_2_ showed a treatment effect while one of the studies without benefits showed adverse effects on markers of inflammation (in rheumatoid arthritis).

Multiple and different markers of inflammation were used in the various studies. CRP was the most commonly used marker; it was used in 26 studies. Vitamin D showed a treatment effect in only 8 of the 26 studies that used CRP, although some of the studies using CRP also used other markers. TNF-α was used as a marker in 16 studies, 4 of which showed a treatment effect. However, studies of highly inflammatory conditions tended to show vitamin D reduced both markers. The marker that showed the most significant decline with vitamin D treatment compared to placebo was the chemokine CXCL9 (P = 5.92 × 10^−^^12^) in Coussens et al.^81^

We found 2 remarkably well conducted RCTs without benefits in low inflammatory conditions (old age and obesity) that met most of our proposed criteria (initial 25(OH)D<50 nmol/L, final 25(OH)D >75 nmol/L; baseline CRP elevated, and physiological doses of vitamin D_3_ used). For example, Bjorkman et al randomized 218 long-term elderly inpatients to receive either placebo or 16,800 IU Q 2 weeks of vitamin D_3_ for 6 months.^46^ Baseline 25(OH)D was 23 nmol/L and final 25(OH)D was 70 nmo/L. Baseline CRP was relatively high at 10.86 mg/L. However, at the end of the trial CRP was not significantly different between groups. Likewise, Warmberget et al^58^ randomized 52 obese subjects to either 7,000 IU of vitamin D_3_/day or placebo. Mean 25(OH)D went from 33 nmol/L at baseline to 113 nmol/L at 26 weeks. Baseline CRP was 7.4 mg/ml. There was no difference between groups at the end of the study.

In the studies we reviewed, vitamin D was studied in sub-physiological and physiological doses.^88^ We could not identify any RCT that used pharmaceutical doses of vitamin D in inflammation although one study used 800 IU/day of calcidol, the equivalent of up to 8,000 IU/day, and showed a treatment effect in rather arcane markers of inflammation in normal subjects.

If vitamin D was an investigational drug, one of the first things pharmaceutical companies would do are dose ranging studies to find the highest dose of the drug that does not cause significant side effects, that is the pharmacological dose. This is important if vitamin D is to be used as a drug, lest a treatment effect be missed with too low of a dose. With the exception of one outlier study, in a meticulous review as noted above, Vieth found the equivalent of 50,000 IU/day did not cause hypercalcemia and was apparently free of serious short-term side effects^.^^89^

While pharmaceutical doses of vitamin D are unknown, in our review we found 5 of the 42 studies used the equivalent of more than 5,000 IU/day of vitamin D. None of the final achieved 25(OH)D concentration exceeded the usual upper limit of normal 25(OH)D ranges (250 nmol/L) so it can be argued these were not supra-physiological doses. Three of the 5 studies using such doses found beneficial effects.

## Conclusions

In highly inflammatory conditions, where markers of inflammation are high at baseline, 6 of 8 RCTs show vitamin D_3_ modestly reduced markers of inflammation with one study of vitamin D_2_ showed an adverse effect.

As far as the chicken and the egg question of does vitamin D lower inflammation or does inflammation lower vitamin D concentration, we conclude RCTs show that improving vitamin D status modestly lowers most markers of inflammation in highly inflammatory conditions. However, it is possible that both mechanisms are at play. That is, vitamin D may decrease inflammation and oxidative stress from inflammation may interfere with the metabolism of vitamin D and thus lower 25(OH)D. The 2 are not mutually exclusive.

After reviewing the above RCTs, we conclude that future RCTs of vitamin D in inflammation and disease should meet the following criteria:

1. Study markedly inflammatory medical conditions;
2. Have elevated baseline markers of inflammation;
3. Have mean baseline 25(OH)D concentration <45 nmol/L;
4. Use doses of vitamin D_3_ sufficient to raise 25(OH)D concentration to >85–100 nmol/L.

Modern studies of the clinical use of physiological doses of vitamin D are relatively rare (around 5,000 IU/day, see above). At least one RCT of physiological doses shows vitamin D may be clinically helpful as add on treatment in multiple sclerosis,^89^ active tuberculosis,^90^ rheumatoid arthritis,^91^ and lupus,^92^ all inflammatory diseases. All clinical treatments decisions are based on a risk/benefit analysis. As the risk of physiological doses of vitamin D is low,^93^ clinicians should consider using physiological doses of vitamin D as add on therapy in inflammatory conditions until RCTs of such doses show it to be of no value.

## Method

To try to answer some of these questions, we attempted to review all the RCTs of vitamin D and inflammatory markers published in the English language as of July, 2014. We searched the National Library of Medicine for key words, “vitamin D” and “inflammation,” and “controlled trial.” We obtained 60 references and then scoured those publications for additional references. For every RCT found, we also searched for similar studies using that Medline search feature. We excluded RCT of 1,25(OH)D_2_D_3_ or its analogs. We then examined these 60 references for outcomes related to inflammation, which reduced the number of studies included to 39. We then examined whether the inflammation outcomes were significant to the p < 0.05 level. In determining the success of the trials in terms of baseline and achieved 25(OH)D concentration, we used the ratio of significant inflammation to total inflammation outcomes for each study. Since we did not follow the rules for systematic reviews, this review should be considered a narrative review.
